# Myxoma Mimic in a Patient With Acute Myeloid Leukemia

**DOI:** 10.7759/cureus.43714

**Published:** 2023-08-18

**Authors:** Natalia Campo-Rivera, Oswaldo Aguilar-Molina, Stephany Barbosa-Balaguera

**Affiliations:** 1 Cardiology, Universidad del Valle, Cali, COL; 2 Cardiology, Hospital Universitario del Valle, Cali, COL

**Keywords:** tumor mimics, acute myeloid leukemia (aml), tricuspid valve regurgitation, cardiac mass tumor, atrial thrombus

## Abstract

Cardiac masses are rare conditions that, depending on their size and location, can cause several cardiac and systemic symptoms. We describe a case of a 21-year-old male with a history of syphilis, pulmonary tuberculosis, and acute myeloid leukemia (AML), in whom a transthoracic echocardiogram assessment was solicited before the initiation of induction chemotherapy. The study revealed a pedunculated, highly mobile mass in the right atrium that protruded to the right ventricle. Surgical resection was performed. During surgery, tricuspid valve perforation was noted and was associated with severe tricuspid valve regurgitation. Histopathological analysis of the resected mass determined that the lesion was a cardiac thrombus.

## Introduction

Cardiac masses are rare conditions (ranging from 0.001% to 0.03% in autopsy series) that, despite being described for the first time in the Middle Ages, still constitute a diagnostic and therapeutic challenge [1]. In 2015, WHO classified cardiac masses as benign tumors, malignant tumors, tumor-like lesions, and pericardial tumors. Neoplastic lesions can be also classified as primary or secondary. Masses may be symptomatic or incidental findings depending on their size and location within the heart [2,3].

Primary lesions are often benign in 90% of the cases. The most frequent benign tumors are myxomas (accounting for almost 50%). Malignant primary tumors are usually sarcomas [3,4]. Secondary (metastatic) cardiac tumors are, by definition malignant, more frequent than primary tumors (ratio 20:1) and may originate from hematogenous or lymphatic dissemination or direct extension usually via superior or inferior vena cava to the right atrium. Metastatic lesions generally arise from melanomas, renal, breast, and lung cancer, as well as from some lymphomas. Tumor-like conditions such as thrombus, pericardial cysts, and Lambl’s excrescences are frequently mistaken for benign or malignant tumors. Cardiac thrombus may be associated with embolic complications [5,6].

In patients with acute myeloid leukemia (AML), a malignant hematopoietic neoplasm characterized by immature myeloid cell proliferation, bone marrow failure, and extramedullary invasion, a cardiac mass can be identified [7]. Diagnostic clues should be used to establish the etiology that may or not be related to this hematopoietic neoplasm. Epidemiological data regarding the most common cardiac mass presentations in patients with acute leukemia is unknown; however, it is worth mentioning that AML can metastasize to the heart and this possibility should be taken into account [8,9].

We report a case of an adult with AML, with an incidental finding of a cardiac mass in the right atrium, in whom transthoracic echocardiography offered important clues but was insufficient to determine its etiology.

## Case presentation

A 21-year-old male with a history of syphilis and pulmonary tuberculosis in the continuation phase presented to the emergency department with fatigue, generalized weakness, and easy bruising. Physical examination revealed pallor, hepatomegaly, and splenomegaly. Cardiac auscultation was normal. There were no signs of systemic or pulmonary congestion. The hemogram revealed leukocytosis, anemia, and thrombocytopenia. The blood smear revealed circulating blasts. Bone marrow aspiration and biopsy were conclusive of AML with minimal differentiation (AML-M0). No chromosomal abnormalities were seen. Central nervous system infiltration was noticed in cerebrospinal fluid analysis.

**Figure 1:**
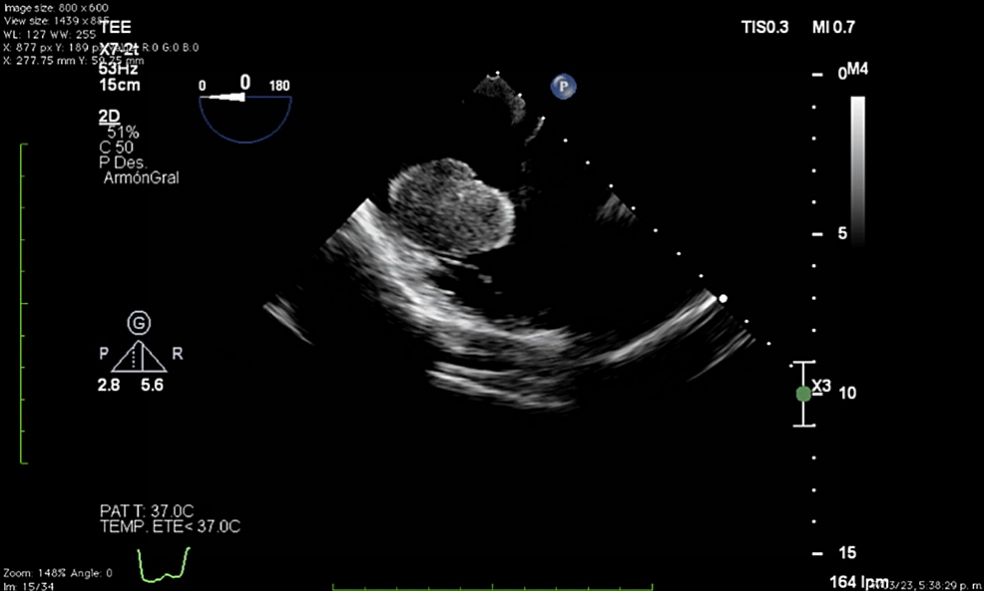
Transthoracic echocardiogram revealed a solid mass measuring 42 mm x 41 mm originating from the lateral wall of the right atrium

**Figure 2:**
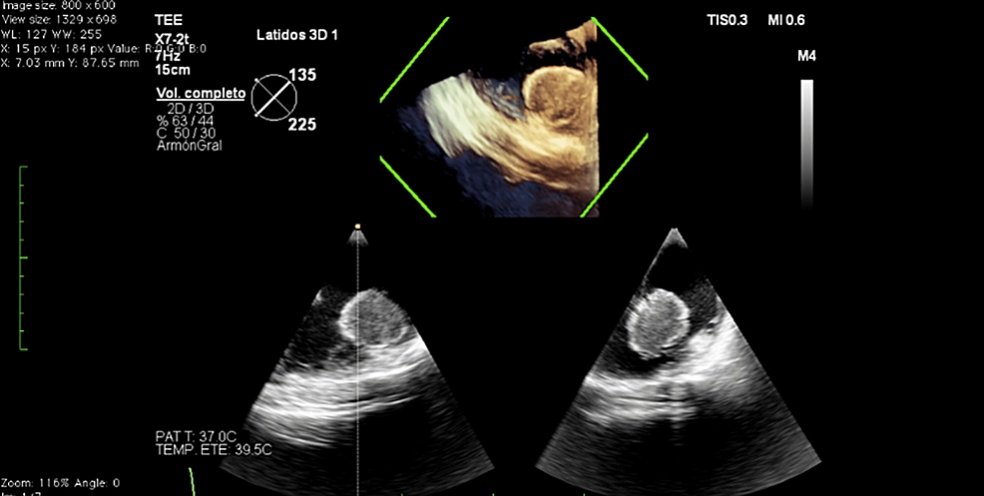
Cardiac mass was connected through a peduncle, with non-homogeneous echogenicity, areas of echolucency, highly mobile, and a systolic excursion to the right ventricle

Before the initiation of fludarabine, cytarabine, idarubicin, and granulocyte colony-stimulating factor (FLAG-IDA) induction, a transthoracic echocardiogram showed a left ventricular ejection fraction of 62%, a global longitudinal strain: -17.1%. A solid mass measuring 42 mm x 41 mm was seen, originating from the lateral wall of the right atrium connected through a peduncle, with non-homogeneous echogenicity, areas of echolucency, highly mobile, and a systolic excursion to the right ventricle (Figures 1-2). The right ventricle and tricuspid valve were not compromised during the initial echocardiographic examination.

**Figure 3:**
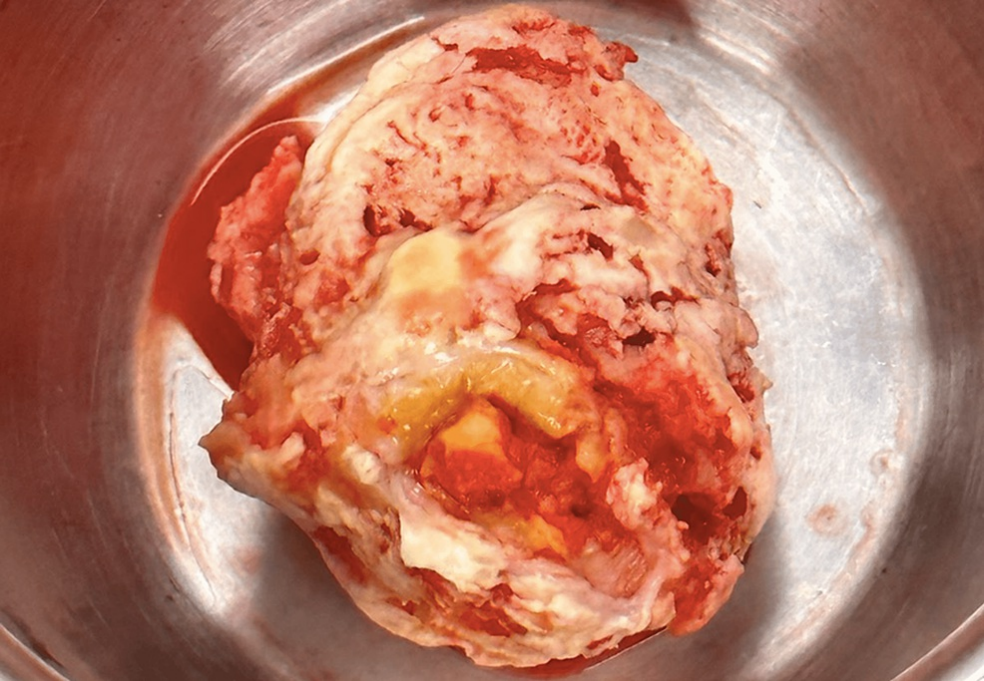
A 5 cm mass was resected. The lesion had a grumous aspect

A surgical resection of the mass was planned after the completion of the induction chemotherapy with FLAG-IDA. The surgical approach was decided due to mass size and systolic excursion to the right ventricle, which confers a risk of mechanical complications. During surgery, a 5 cm mass was seen in the right atrium. The lesion had a grumous aspect and was adhered through a peduncle to the posterior aspect of the right atrium and to the tricuspid leaflets (Figure 3). The mass did not extend to the inferior or superior vena cava.

**Figure 4:**
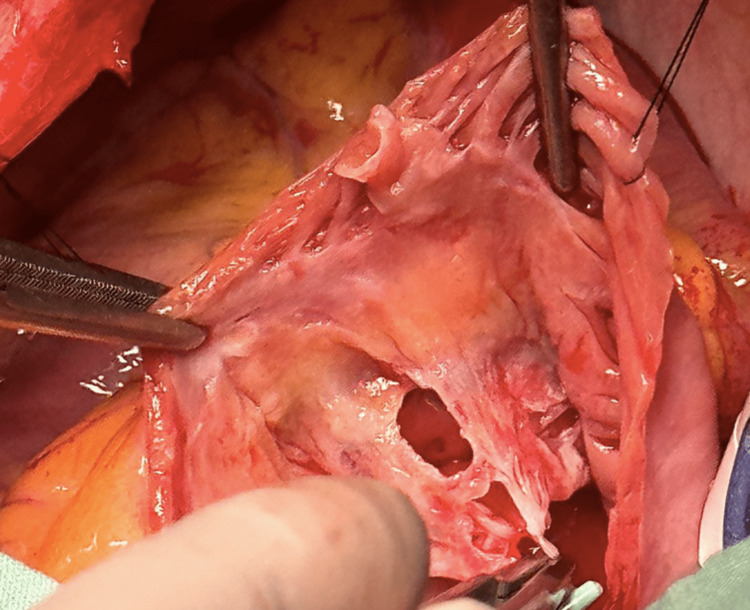
When the tricuspid valve was exposed, it was recognized that the valve had multiple perforations not seen during echocardiographic assessment

When the tricuspid valve was exposed, it was recognized that the valve had multiple perforations not seen during echocardiographic assessment (Figure 4).

The perforations were conditioning a severe tricuspid regurgitation. The surgical team determined that the valvular destruction noted implied technical difficulties for repair using a pericardial patch; therefore, a tricuspid valve replacement was performed with a Medtronic Hancock II #33 valve (Medtronic, Dublin, Ireland).

Histopathological examination of the mass revealed a thrombus adhered to fibroconnective tissue. The surface had calcifications and acute and chronic inflammatory infiltrate. No neoplastic cells were identified in the sample. After surgery, the patient has remained cardiovascular asymptomatic and is currently anticoagulated with warfarin without any complications.

## Discussion

Cardiac masses may be symptomatic or incidental findings depending on their size and location within the heart. Symptoms can be systemic, such as paraneoplastic syndromes, arthralgias, weight loss, and fever. Cardiac symptoms include dyspnea, syncope, and chest discomfort. The mass effect may result in arrhythmias, interfere with valvular function causing regurgitation, or compromise the pericardium leading to the development of pericardial effusion with or without associated tamponade. Another complication of cardiac masses is systemic and/or pulmonary embolism (PE) [10].

Etiological identification of cardiac masses should start with the determination of symptoms, epidemiologic likelihood, and clinical probability based on the location of the tumor and echocardiographic clues [10].

The patient presented without apparent symptoms. In retrospect, it is possible that cardiac symptoms were present but were difficult to assess due to both AML and pulmonary tuberculosis, which may present with fatigue, shortness of breath, and exercise intolerance [11].

Regarding the location, cardiac masses found on the right include myxoma, lymphoma, metastasis, lipoma, angiosarcoma, thrombus, and cardiac involvement of AML [7,9,10]. Since the patient had an established oncological diagnosis, it was presumed that the lesion had a malignant origin [10]. The definite diagnosis of cardiac thrombus was made by histopathological examination.

The incidence of thrombosis in AML among different studies ranges from 2.09% to 5.4%. It develops due to several mechanisms including the increase in procoagulant particles, reduced levels of anticoagulant factors, platelet activation, and a decrease in fibrinolytic activity [12]. Thrombosis in AML usually occurs in the lungs or in the upper or lower limbs [13]. The incidence of cardiac thrombus in AML has not been established.

Cardiac thrombus in cardiac imaging is characterized by avascular tissue (best identified by contrast-enhanced imaging). It is a l-w signal-intensity image that is surrounded by high-signal-intensity tissues such as surrounding myocardium and/or cavity blood. A mass that is attached to an area of abnormal wall motion, such as dyskinesis or akinesis, should also raise clinical suspicion [14-16].

The incidence of right atrium thrombus is unknown. Patients at risk are those with right-sided pacemaker leads, mechanical valves, central venous lines, and/or ventricular or atrial septal closure devices [17,18]. The presence of right atrium thrombus increases the risk of PE and has been described in 10% of patients with PE and in 6.5% of patients with PE confirmed by autopsy [19].

Cardiac thrombus was not the most suspected etiology in this case, owing to the facts that it was not attached to an area of abnormal wall motion, the right atrium is an unusual location, the mass was pedunculated, and the lesion perforated multiple locations of the tricuspid valve [1].

In general terms, tricuspid regurgitation has a secondary etiology in more than 90% of the cases, and it may be a consequence of volume and/or pressure overload that mediates right ventricular dilatation, or due to dilated right atrium and tricuspid annulus in patients with chronic atrial fibrillation. Primary tricuspid regurgitation, as seen in this patient, is often due to infective endocarditis, carcinoid syndrome, myxomatous disease, rheumatic heart disease, thoracic trauma, iatrogenic valve damage, endomyocardial fibrosis, and congenital valve dysplasia [20].

To our knowledge, this is the first reported case of a cardiac thrombus occurring in a patient with AML and a structurally normal heart that, despite being adhered to the posterior aspect of the right atrium, due to its big size, high mobility, and systolic excursion to the right ventricle, perforated the tricuspid valve leading to severe tricuspid primary regurgitation.

## Conclusions

Cardiac masses are infrequent conditions that are usually of benign etiology. In order to establish the origin, clinicians should explore symptoms, past medical history, location, and echocardiographic clues. A definite diagnosis is made with histopathological examination. Complications of a cardiac mass, which include valvular damage, depend mainly on the size and location of the lesion.

## References

[1] Mankad R, Herrmann J (2016). Cardiac tumors: echo assessment. Echo Res Pract.

[2] (2015). WHO classification of tumours of the lung, pleura, thymus and heart. Heart 4th edn (World.

[3] Bussani R, Castrichini M, Restivo L (2020). Cardiac tumors: diagnosis, prognosis, and treatment. Curr Cardiol Rep.

[4] Ekmektzoglou KA, Samelis GF, Xanthos T (2008). Heart and tumors: location, metastasis, clinical manifestations, diagnostic approaches and therapeutic considerations. J Cardiovasc Med (Hagerstown).

[5] Paraskevaidis IA, Michalakeas CA, Papadopoulos CH, Anastasiou-Nana M (2011). Cardiac tumors. ISRN Oncol.

[6] Basso C, Rizzo S, Valente M, Thiene G (2016). Cardiac masses and tumours. Heart.

[7] Zanetti C, Inciardi RM, Benedetti F, Rossi A (2018). Right cardiac chambers' involvement as the first manifestation of recurrent complex karyotype acute myeloid leukemia. J Cardiovasc Echogr.

[8] Hudzik B, Miszalski-Jamka K, Glowacki J (2015). Malignant tumors of the heart. Cancer Epidemiol.

[9] De Lazzari M, Fedrigo M, Marra MP (2015). Relapsing leukemia infiltrating the heart. Circ Heart Fail.

[10] Tyebally S, Chen D, Bhattacharyya S (2020). Cardiac tumors: JACC cardiooncology state-of-the-art review. JACC CardioOncol.

[11] Duan WB, Gong LZ, Jia JS (2017). Clinical features and early treatment effects in intermediate risk and poor risk acute myeloid leukemia with EVI1 positive [Article in Chinese]. Beijing Da Xue Xue Bao Yi Xue Ban.

[12] Olivi M, Di Biase F, Lanzarone G (2023). Thrombosis in acute myeloid leukemia: pathogenesis, risk factors and therapeutic challenges. Curr Treat Options Oncol.

[13] Vu K, Luong NV, Hubbard J (2015). A retrospective study of venous thromboembolism in acute leukemia patients treated at the University of Texas MD Anderson Cancer Center. Cancer Med.

[14] Srichai MB, Junor C, Rodriguez LL (2006). Clinical, imaging, and pathological characteristics of left ventricular thrombus: a comparison of contrast-enhanced magnetic resonance imaging, transthoracic echocardiography, and transesophageal echocardiography with surgical or pathological validation. Am Heart J.

[15] Weinsaft JW, Kim HW, Shah DJ (2008). Detection of left ventricular thrombus by delayed-enhancement cardiovascular magnetic resonance prevalence and markers in patients with systolic dysfunction. J Am Coll Cardiol.

[16] Weinsaft JW, Kim RJ, Ross M (2009). Contrast-enhanced anatomic imaging as compared to contrast-enhanced tissue characterization for detection of left ventricular thrombus. JACC Cardiovasc Imaging.

[17] Yilmaz M, Gurlertop Y, Erdogan F (2003). Right atrial thrombus following closure of an atrial septal defect. Heart.

[18] Burns KE, McLaren A (2009). Catheter-related right atrial thrombus and pulmonary embolism: a case report and systematic review of the literature. Can Respir J.

[19] Ogren M, Bergqvist D, Eriksson H, Lindblad B, Sternby NH (2005). Prevalence and risk of pulmonary embolism in patients with intracardiac thrombosis: a population-based study of 23 796 consecutive autopsies. Eur Heart J.

[20] Vahanian A, Beyersdorf F, Praz F (2022). 2021 ESC/EACTS Guidelines for the management of valvular heart disease. Eur Heart J.

